# Mobile phone text message reminders of antipsychotic medication: is it time and who should receive them? A cross-sectional trust-wide survey of psychiatric inpatients

**DOI:** 10.1186/1471-244X-14-15

**Published:** 2014-01-22

**Authors:** Katherine Bogart, Sook Kuan Wong, Christine Lewis, Anthony Akenzua, Daniel Hayes, Athanasios Prountzos, Chike Ify Okocha, Eugenia Kravariti

**Affiliations:** 1 Department of Psychosis Studies, Institute of Psychiatry at the Maudsley, King’s College London, De Crespigny Park, Denmark Hill, London SE5 8AF, UK; 2 Wandsworth Psychological Therapies and Wellbeing, Springfield University Hospital, 61 Glenburnie Road, London SW17 7DJ, UK; 3 Bexley Crisis Resolution and Home Treatment Team, Erith Centre, Park Crescent, Erith DA8 3EE, UK; 4 Oxleas NHS Foundation Trust, Pinewood House, Pinewood Place, Dartford, Kent DA2 7WG, UK

**Keywords:** Antipsychotics, Medication adherence, Electronic reminders, SMS, Mobile phone, Inpatients

## Abstract

**Background:**

Poor adherence to antipsychotic medication is a widespread problem, and the largest predictor of relapse in patients with psychosis. Electronic reminders are increasingly used to improve medication adherence for a variety of medical conditions, but have received little attention in the context of psychotic disorders. We aimed to explore the feasibility and acceptability of including short message service (SMS) medication reminders in the aftercare plan of service users discharged from inpatient care on maintenance antipsychotic medication.

**Methods:**

We conducted a cross-sectional, trust-wide survey in the inpatient units of the Oxleas National Health Service (NHS) Foundation Trust in the UK between June 29 and August 3, 2012. Using a self-report questionnaire and the Drug Attitude Inventory, we examined inpatient attitudes towards antipsychotic drugs, past adherence to antipsychotic medication, frequency of mobile phone ownership, and interest in receiving SMS medication reminders upon discharge from the ward. Predictors of a patient’s interest in receiving electronic reminders were examined using simple logistic regression models.

**Results:**

Of 273 inpatients, 85 met eligibility criteria for the survey, showed decisional capacity, and agreed to participate. Of the 85 respondents, over a third (31-35%) admitted to have forgotten to take/collect their antipsychotic medication in the past, and approximately half (49%) to have intentionally skipped their antipsychotics or taken a smaller dose than prescribed. Male patients (55%), those with negative attitudes towards antipsychotics (40%), and those unsatisfied with the information they received on medication (35%) were approximately 3 to 4 times more likely to report past intentional poor adherence. The large majority of respondents (80-82%) reported having a mobile phone and knowing how to use SMS, and a smaller majority (59%) expressed an interest in receiving SMS medication reminders after discharge. No variable predicted a patient’s interest in receiving electronic reminders of antipsychotics.

**Conclusions:**

Automatic SMS reminders of antipsychotic medication were acceptable to the majority of the survey respondents as an optional service offered upon discharge from inpatient care. Automatic electronic reminders deserve further investigation as a flexible, minimally invasive, cost-effective and broadly applicable tool that can potentially improve antipsychotic adherence and clinical outcomes.

## Background

Psychoses are severe disorders of thinking, perception and emotions, which affect 50 per 100,000 individuals in the UK every year [1]. Episodes of psychosis, together with dementia and quadriplegia, are ranked in the highest disability “class” by health professionals [2], and occur in the context of several psychiatric disorders - mainly schizophrenia, but also depression, bipolar illness and substance abuse disorders [3]. The substantial cost of schizophrenia to UK society, estimated to approach £7 billion in 2004/05 [4], is largely accounted for by inpatient stays, pharmacological and psychosocial interventions and loss of financial opportunity [5].

Much of the burden psychotic disorders place on patients, carers, the health service and society, is the result of relapses, which typically disrupt psychosocial and vocational adjustment, and increase the risk of hospitalisation and suicide [6]. Antipsychotic medication, the first-line treatment for psychosis [7], can be highly effective in preventing relapse [8], particularly after the first episode [9]. Unfortunately, a substantial proportion of patients (35-75%) discontinue their antipsychotic medication within 1-2 years of follow-up, a further subgroup are only partially adherent, and only a minority (8-33%) are fully adherent to treatment [10-14]. Poor adherence to antipsychotic medication is the largest predictor of relapse in patients with psychosis [15].

Treatment discontinuation and poor adherence to antipsychotics can result from several factors, including lack of insight, concern about stigma, persistent symptoms or side effects, lack of routines, cognitive deficits, poor therapeutic alliance, and lack of family support [16]. Negative attitudes towards antipsychotic medication are the strongest predictor of treatment discontinuation, particularly in first-episode schizophrenia [17-21]. In general, while negative attitudes usually contribute to a patient’s conscious choice to skip or discontinue medication in various medical contexts, time-keeping difficulties and forgetfulness can play a critical role in unintentional non-adherence [22,23].

Electronic reminders (automatically sent reminders without personal contact between the healthcare provider and the patient) are now increasingly used to improve adherence to long-term treatment for a variety of medical conditions [24]. The short-term effectiveness of these paradigms, especially short message service (SMS) reminders, is receiving increasing support, although their long-term effects are yet to be established [24]. To date, 3 studies have examined the value of SMS interventions in improving medication adherence in patients with psychosis [25-27], and 2 reported positive findings [26,27]. As these studies were conducted in Spain, the US and the Netherlands, it is unclear how cross-national differences in attitudes towards mobile technology or e-health applications, in demographic factors, or in infrastructure capacity may influence the applicability of the respective findings to the UK.

Several questions are worth exploring before piloting electronic systems of antipsychotic medication reminders for users of psychiatric services in the UK. For example, what percentage of patients who are regularly prescribed oral antipsychotics own a mobile phone, know how to use SMS and are willing to receive medication reminders? What percentage of this population report forgetting to take their antipsychotic medication in the community (and hence would most likely benefit from electronic reminders)? What factors predict a patient’s willingness to receive SMS antipsychotic medication reminders?

We report on a Trust-Wide survey conducted in the inpatient units of the Oxleas National Health Service (NHS) Foundation Trust, which addressed the above questions. The general aim of the study was to explore the feasibility and acceptability of including SMS medication reminders in the standard aftercare plan of users who are discharged from inpatient psychiatric units on maintenance antipsychotic medication.

## Methods

### Participants

**Figure 1:**
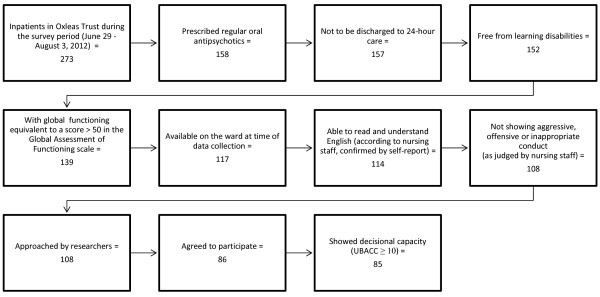
Process of sample derivation.

Over the survey period (June 29-August 3, 2012) a total of 273 adult patients received psychiatric inpatient services at the Oxleas National Health Service (NHS) Foundation Trust (in the Greenwich, Bromley and Bexley inpatient units, comprising eight acute wards). Of these, 108 patients met general eligibility criteria for the study (see below) and were invited to participate, 86 agreed to take part, and 85 demonstrated decisional capacity, making up the final study sample. All participants met the following inclusion criteria: 1) They were receiving regular doses of oral antipsychotic medication, which would also be prescribed upon discharge, 2) they demonstrated psychological, social and occupational functioning equivalent to a score of >50 in the Global Assessment of Functioning (GAF) Scale [28] (indicating less than severe difficulties), 3) they were able to read and understand English (according to nursing staff and self-report), 4) they demonstrated decisional capacity based on the administration of the University of California, San Diego Brief Assessment of Capacity to Consent (UBACC) scale [29], and 5) they provided written informed consent for the study. Patients with diagnoses of learning disabilities and those who would be discharged into 24-hour care residential homes were excluded, as these groups were likely to rely on caregivers for receiving their medication, and were thus unlikely to benefit from text message reminders. We also excluded patients considered to be unsuitable for the study by the ward staff (e.g. those with offensive, aggressive or inappropriate conduct). The patients’ eligibility for the study was established based on a variety of sources, including drug charts, schedules routinely administered in the clinical setting (GAF) [28], the study questionnaires (see below), the electronic clinical records system of the Oxleas NHS Foundation Trust (RiO), and consultations with mental health practitioners in charge of the patients’ care. The process of deriving the study sample is summarised in Figure 1.

The study was approved by the Oxleas Research and Development office, which advised that the investigation was a service evaluation and did not require ethical approval.

### Assessments

The University of California, San Diego Brief Assessment of Capacity to consent (UBACC) scale [29] was used to assess the prospective participants’ level of comprehension of the study, and capacity to consent. The UBACC is a flexible instrument with brief administration time (approximately 5 minutes), which can be tailored to the specific research protocol of each study [29].

A 10-item self-report questionnaire was used to examine 1) the feasibility and acceptability of using SMS reminders of antipsychotic medication in patients with severe mental illness after they are discharged from an acute psychiatric unit, and 2) patterns of past adherence to antipsychotic medication. The instrument (items shown in Table 1), was adapted from a survey of adherence to antidepressant medications in community settings [30].

**Table 1:** Participant responses to the survey questionnaire

	**Participants (n = 85)**	
	**Yes**	
**Questions**	**N**	**%**^**1,2**^
1) Do you have a mobile telephone?	70	82
2) Can you use the text messaging services?	68	80
**Before coming to this ward:**		
3) Were you ever prescribed antipsychotic medications **(if NO, go to question 8)**	74	87
4) Did you ever forget to collect your antipsychotic prescription?^1^	26	35
5) Did you ever forget to take your medication?^1^	23	31
6) Did you ever intentionally skip your antipsychotic medication or take less than the prescribed dose?^1^**(if NO, go to question 8)**	36	49
7) If YES to question 6, what were your reasons (TICK all that apply)^**2**^:		
i. I did not like the side effects	18	50
ii. I thought that a smaller dose would do fine	15	42
iii. I was trying to cut down on the number of tablets I took	14	39
iv. I did not think they actually worked that well	14	39
v. I felt better without them	10	28
vi. I was not convinced I had an illness	14	39
vii. I did not have symptoms at the time	18	50
vii. Other (please explain):		
8) Do you feel you receive enough information about antipsychotic medication?	55	65
**After you have been discharged:**		
9) Would you be interested in receiving text message reminders from the Trust to help you to remember to take your antipsychotic medications?	50	59

^1^The percentages of respondents who answered YES to questions 4, 5, 6 refer to the sub-sample of 74 respondents who answered YES to question 3.

^2^The percentages of respondents who answered YES to items i - vii of question 7 refer to the sub-sample of 36 respondents who answered YES to question 6.

As a potential predictor of medication adherence and of the interest in receiving text-message reminders of antipsychotic medication, attitudes to antipsychotic treatment were assessed using the short form of the Drug Attitude Inventory (DAI-10) [31]. This is a simple and easy-to-use self-report instrument with good psychometric properties, which assesses a unique clinical dimension relevant to non-adherence [17]. A positive sum of scores indicates a positive subjective response to medication and a negative sum of scores indicates a negative subjective response.

### Data analysis

The data were analysed using IBM SPSS Statistics 20 and STATA 11. Descriptive statistics were used to explore the frequency of the following characteristics in the sample: Mobile phone ownership, knowing how to use text-messaging, interest in receiving automatic medication reminders after hospital discharge, poor past adherence to medication, and positive/negative drug attitudes. Potential predictors of a patient’s interest in receiving text message reminders of antipsychotic medication were explored in a series of simple logistic regression models. The following predictors were analysed: Individual demographic and clinical characteristics (as shown in Table 2), mobile phone ownership, past intentional or unintentional non-adherence to antipsychotic medication, satisfaction with the medication information received by the health services, and attitudes towards antipsychotic treatment.

**Table 2:** Demographic and clinical characteristics of participants

		**Participants (n = 85)**	
**Variables**		**N**	**%**^**1**^
**Gender**			
	Female	38	45
	Male	47	55
**Age (Years)**			
	18-35	48	57
	36-50	30	35
	51-67	7	8
**Ethnicity**			
	White	58	68
	Black	10	12
	Other	15	18
**Employment status**			
	Working or studying	15	18
	Not working	65	76
**Marital status**			
	Has partner	25	29
	No partner	60	71
**Diagnosis (disorder)**			
	Nonaffective	32	38
	Affective	16	18
	Substance induced/organic	23	27
	Other	14	17
**Antipsychotics medications**^**2**^			
	Risperidone	18	21
	Quetiapine	29	34
	Clozapine	8	9
	Olanzapine	24	28
	Other	6	8

^1^The cumulative percentages for ‘Ethnicity’ and ‘Employment Status’ are 98% and 94%, respectively, due to missing values.

^2^Daily dosage of prescribed antipsychotic (% of patients): 0.5-30 mg (47%), 50-250 mg (8%), 300-800 mg (30%).

## Results

The demographic and clinical characteristics of the analytic sample (n = 85) are shown in Table 2. A summary of the survey findings is presented in Table 1. The results of the regression analyses in relation to potential predictors of a patient’s willingness to receive electronic reminders are shown in Table 3.

**Table 3:** Predictors of interest in receiving text message reminders of antipsychotic medication

		**LR chi2**	**OR**	**95% CI**		**P**
				**From**	**To**	
**Gender**		0.53				0.47
	Female		1.00			
	Male		0.72	0.30	1.73	
**Age**		5.59				0.06
	18–35		1.00			
	36–50		0.45	0.18	1.16	
	51–67		0.18	0.03	1.05	
**Ethnicity**		3.62				0.16
	White		1.00			
	Black		0.26	0.06	1.12	
	Other		0.92	0.29	2.93	
**Employment**		0.01				0.93
	Working or studying		1.00			
	Not working		0.95	0.30	2.99	
**Marital Status**		0.09				0.76
	Has partner		1.00			
	No partner		1.17	0.44	3.12	
**Diagnosis (disorder)**		1.19				0.76
	Nonaffective		1.00			
	Affective		1.10	0.27	4.55	
	Substance induced/organic		1.60	0.3	8.49	
	Other		0.60	0.12	2.97	
**Type of antipsychotic agent**		0.62				0.96
	Risperidone		1.00			
	Quetiapine		1.31	0.40	4.32	
	Clozapine		0.80	0.15	4.24	
	Olanzapine		1.12	0.33	3.85	
	Other		1.60	0.23	11.08	
**Do you have a mobile telephone?**		0.47				0.50
	No		1.00			
	Yes		0.67	0.21	2.15	
**Did you ever forget to take your medication?**		0.06				0.80
	No		1.00			
	Yes		0.88	0.33	2.38	
**Did you ever intentionally skip … or take less than the prescribed dose?**		0.05				0.83
	No		1.00			
	Yes		1.11	0.44	2.79	
**Do you feel you receive enough information … medication?**		0.39				0.53
	No		1.00			
	Yes		0.74	0.30	1.87	
**Drug Attitutes (DAI score)**		0.20				0.65
	Positive		1.00			
	Negative		0.82	0.34	1.97	

Of the 85 participants, 82% reported having a mobile phone. Knowing how to use SMS was reported by 80% of the total analytic sample and 90% of those who had a mobile phone. Similar percentages in the total analytic sample (59%) and in the subgroup of mobile-phone owners (57%) expressed an interest in receiving SMS medication reminders after being discharged from the ward (Table 1).

Of the 74 inpatients who reported having been prescribed antipsychotic agents in the past, just over a third (31-35%) reported experiences of unintentional non-adherence, and approximately half (49%) admitted to have intentionally skipped their antipsychotic medication or taken a smaller dose than prescribed. The most commonly stated reasons for intentional non-adherence were ‘side effects’ (50%), ‘not having symptoms at the time’ (50%), and ‘thinking that a smaller dose would do fine’ (42%) (Table 1). Of the subgroup of unintentional non-adherers, 56% expressed an interest in receiving SMS medication reminders after being discharged from the ward.

No demographic or clinical variable predicted interest in receiving SMS medication reminders, although age showed a trend towards significance (Table 3). Mobile phone ownership, past adherence to antipsychotic medication, satisfaction with medication information, and attitudes towards antipsychotic treatment also failed to predict interest in receiving SMS medication reminders (Table 3).

Of the 85 participants, those with negative attitudes towards antipsychotic medication (40%), those who perceived the information they received on treatment as insufficient (35%) and males (55%) were approximately 3 to 4 times more likely to report having intentionally skipped their antipsychotic medication in the past (negative attitudes: OR = 4.00, 95% CI 1.50 to 10.66, p < 0.01; insufficient information: OR = 2.79, 95% CI 1.07 to 7.29, p < 0.05; male gender: OR = 2.95, 95% CI 1.14 to 7.63, p < 0.05). None of the three variables was a significant predictor of unintentional non-adherence (OR = 0.75-1.49, p > 0.10).

## Discussion

Our trust-wide survey at the Oxleas NHS Foundation Trust demonstrated that approximately 60% of in-patients with psychotic disorders have the resources and willingness to benefit from SMS-based interventions for increasing medication adherence. More than three quarters of the respondents reported to own a mobile phone (82%) and be able to use SMS (80%), while 59% expressed an interest in receiving electronic prompts from the Trust to remind them to take their oral antipsychotics after discharge.

In line with previous findings [10-14], the prevalence of self-reported poor adherence to antipsychotic medication among the survey respondents was substantial. Over a third (31-35%) reported to have previously forgotten to take or collect their antipsychotic medication, and approximately half (49%) to have intentionally skipped, or taken less than, the prescribed dose.

There were no demographic or clinical characteristics that predicted a patient’s interest in receiving electronic medication reminders. Similarly, owning a mobile phone, previous levels and patterns of adherence to medication, level of satisfaction with the medication information received from the services, and attitudes towards antipsychotic treatment were not significantly associated with a patient’s interest in receiving reminders.

Our findings suggest that, regardless of demographic and diagnostic characteristics, attitudes towards medication, and patterns of past adherence, the majority of patients with psychotic disorders are likely to respond positively to an offer of electronic reminders of antipsychotic medication as an optional care provision after discharge from an inpatient ward. Therefore, SMS prompts of antipsychotic medication show promise as a low-cost intervention, which could potentially result in large-scale clinical and economic benefits through intervening in the adverse pathway from medication non-adherence to relapse to inpatient admission [7-9,15]. Furthermore, the prominence of both intentional and involuntary factors in the aetiology of non-adherence, suggests that personalised, needs-based approaches, which variably incorporate reminders and/or motivational content, might be optimally suited for maximising the benefits of SMS-based interventions.

Male gender, negative attitudes towards antipsychotic medication and lack of satisfaction with the information received on medication were significantly associated with an approximately 3- to 4- fold increase in risk for intentional non-adherence in the present study. These findings suggest a critical need for encouraging positive attitudes towards antipsychotic medication, especially among male patients. In addition, it is important for patients to receive sufficient information about antipsychotic treatment. Well-informed patients are more likely to have insight into their illness, recognise the importance of treatment adherence [17,32-34], and develop therapeutic alliance with service providers.

Adopting SMS technologies is increasingly common and affordable in the UK. National figures show a rapid increase in mobile phone ownership from approximately 79% in 2010 to 91% in 2011 [35,36]. This trend is also present among patients with severe mental illness. Compared to the prevalence of mobile phone ownership in the present study (82%), only 62% of psychiatric inpatients in the Oxleas Trust were reported to own a mobile phone in an earlier survey by our group [37]. In addition to the dramatic increase in mobile device use and infrastructure capacity in the UK, these figures suggest that the prevalence of mobile phone ownership among patients with severe mental illness is lower than in the general population. This observation reflects global trends. For example, a 2011 survey of 1,592 individuals with serious mental illness (SMI) in the U.S. reported a 72% prevalence of mobile device ownership among people with SMI, which was approximately 12% lower than found in the general adult population [38]. The most frequently reported barrier to mobile device ownership was cost [38], reflecting the well-established relationship between poverty and severe mental illness [39]. However, less than a quarter of mobile device non-users in the U.S. sample indicated a lack of interest, and even fewer said they did not know how to use a mobile device [38], suggesting a narrow influence of such barriers on patients with severe mental illness.

Our findings add to a growing body of evidence highlighting the value of treatment-supporting technologies in improving medication adherence and other clinical outcomes in severe mental illness, including internet-based interventions [40], short-message-service technologies [24,41] and electronic diaries [42]. It is important to emphasize that any recommendations for addressing medication non-adherence in the context of psychotic disorders should acknowledge the extensive evidence base demonstrating that a combination of adjunctive educational, behavioural, and affective strategies is likely to prove more effective than any intervention used in isolation [43]. It is further recommended that the effect of interventions on medication adherence be considered alongside other important outcome parameters, such as quality of life [44].

Our study findings should be considered in the context of potential imitations. As our aim was to establish the acceptability of electronic medication reminders at the point of discharge from inpatient care, our survey focused on psychiatric inpatients. However, individuals admitted to a mental health unit are typically very unwell [45]. We addressed this inherent limitation by introducing a variety of inclusion and exclusion criteria in our selection strategy, including functional and decisional capacity in our study inclusion criteria, and learning disabilities, discharge into 24-hour residential care, and clinician-judged ‘unsuitability’ for the study (offensive, aggressive or inappropriate conduct) in our exclusion criteria. This approach may have introduced bias into the study sample, and reduced the relevance and applicability of our findings to patients at the lower end of the functional spectrum (who may differ from the study participants during both acute and sub-acute phases). In addition, we limited our inclusion strategy to patients who can read and understand English. This selection strategy reduced the generalizability of our findings to patients with a migrant status. It would thus be informative to compare the present findings with those of a future survey of psychiatric outpatients on regular antipsychotic prescriptions. Other potential limitations of the study include the unmeasured social desirability effect, which may have inflated the percentage of participants responding affirmatively to some survey items; the assumption that the survey respondents knew the answers to all the questions (however, it is possible that some participants were not able to accurately recall, and/or correctly classify as ‘antipsychotics’, some of the drugs they were prescribed in the past); the relatively narrow range of items included in the core questionnaire, which excluded potentially interesting questions about the use of SMS in the target population (for example, how extensively patients used SMS; for what purposes; what kinds of messages they might like to receive, how the reminders could best address their needs); the relatively small size of the survey sample, which may have failed to adequately capture the range and proportions of the different psychotic disorders in the clinical population; and the limitation of conducting the survey at a single institution, potentially reducing the generalizability of findings. Finally, the prevalence of self-reported intentional and unintentional non-adherence was a secondary focus of the present survey, but self-reports have been criticised for under-estimating the true extent of poor adherence [46], and, therefore, ought to be interpreted with caution.

## Conclusion

Our findings suggest that an optional service of automatic SMS reminders of antipsychotic medication, offered upon discharge from an inpatient unit, is acceptable to the majority of psychiatric patients who are prescribed oral antipsychotic medication, regardless of their demographic and diagnostic characteristics, attitudes towards medication and levels of past adherence. The prospect of a flexible, minimally invasive, cost-effective and broadly applicable tool that can potentially improve adherence to antipsychotic treatment is exciting. The results of the present survey should be extended by future pilot and definitive trials investigating how the reminder service could work in practice, and if it improves medication adherence and clinical outcomes. Our study findings further suggest that improving adherence to antipsychotic treatment should critically incorporate strategies for improving attitudes towards, and dissemination of information on, antipsychotic medication.

## Abbreviations

GAF: Global assessment of functioning; UBACC: University of California, San Diego brief assessment of capacity to consent; DAI: Drug attitude inventory; SMS: Short messaging service.

## Competing interests

The authors declare that they have no competing interests.

## Authors’ contributions

CIO conceived the idea for the study and EK the initial design and data analytical approach. In collaboration with KB, SKW, CL and AA, the quantitative design was refined and further developed. KB and SKW participated in all steps of quantitative study procedures, conduction, data verification, analyses and interpretation. KB, SKW and EK had full access to all of the aggregated data for the study and were responsible for initial interpretation. CL, AA, DH, AP and CIO contributed to reviewing and augmenting the interpretation of quantitative findings following the first and revised manuscript drafts. All authors have contributed critical review and feedback and approved the final version of the manuscript.

## Authors’ information

Katherine Bogart is a trainee low-intensity therapist at South West London and St George’s Mental Health Trust, with a specific interest in chronic illness and treatment adherence.

Sook Kuan Wong is a Psychology graduate with research interests in e-health applications to severe mental illness.

Christine Lewis is an associate specialist in Psychiatry working within a crisis resolution and home treatment team.

Anthony Akenzua is a consultant psychiatrist and clinical director of adult acute mental health services for Oxleas NHS Foundation Trust.

Daniel Hayes is a PhD student at the Institute of Psychiatry, King’s College London, with a broad research interest in e-health applications to psychosis.

Athanasios Prountzos is a research psychologist with interests in e-health applications to severe mental illness.

Chike Ify Okocha is a consultant psychiatrist and medical director for Oxleas NHS Foundation Trust with research interests in psychosis, service design and improvements in quality of clinical care.

Eugenia Kravariti is a Lecturer in Psychosis Studies at the Institute of Psychiatry, King’s College London, with research interests in the neuropsychology of psychosis and in e-health applications to severe mental illness.

Katherine Bogart and Sook Kuan Wong are joint first authors.

## Pre-publication history

The pre-publication history for this paper can be accessed here:

http://www.biomedcentral.com/1471-244X/14/15/prepub
